# EGF Receptor Transactivation by Endothelin-1 Increased CHSY-1 Mediated by NADPH Oxidase and Phosphorylation of ERK1/2

**DOI:** 10.22074/cellj.2021.7392

**Published:** 2021-10-30

**Authors:** Hossein Babaahmadi-Rezaei, Alireza Kheirollah, Mojtaba Rashidi, Faezeh Seif, Zahra Niknam, Masoumeh Zamanpour

**Affiliations:** 1. Hyperlipidemia Research Center, Department of Clinical Biochemistry, Faculty of Medicine, Ahvaz Jundishapur University of Medical Sciences, Ahvaz, Iran; 2. Cellular and Molecular Research Center, Department of Clinical Biochemistry, Faculty of Medicine, Ahvaz Jundishapur University of Medical Sciences, Ahvaz, Iran.; 3. Shoushtar Faculty of Medical Sciences, Shoushtar, Iran

**Keywords:** CHSY1 Enzyme, Endothelin-1, Epidermal Growth Factor, NADPH Oxidase

## Abstract

**Objective:**

Growth factors [transforming growth factor-β (TGF-β), epidermal growth factor (EGF), endothelin-1 (ET- 1)] stimulate proteoglycan synthesis resulting in retention and accumulation of low density lipoprotein (LDL) in vessel intima and leading to atherosclerosis development. This study investigated the role of ET-1 on the expression of CHSY1, proteoglycan synthesizing enzyme, through both EGF and TGF-β receptor transactivation in human vascular smooth muscle cells (VSMCs). Also, we explored the involvement of NADPH oxidase (NOX), an important intermediate of redox signaling, in ET-1 transactivated EGF receptor (EGFR) through endothelin receptors.

**Materials and Methods:**

In this experimental study, phosphorylated ERK1/2 and CHSY1 protein levels in the human VSMCs were measured by Western blot analysis using anti phospho-ERK1/2 (Thr202/Tyr204) and anti CHSY1 antibodies.

**Results:**

ET-1 (100 nM) and EGF (100 ng/ml) stimulated ERK1/2 phosphorylation and inhibited in the presence of bosentan (ET receptor inhibitor), AG1478 (EGFR inhibitor), and DPI (NOX antagonist). Also, ET-1 treatment increased CHSY1 enzyme level; this response was suppressed by bosentan, AG1478, DPI, and SB431542, TGF-β receptor antagonist. This study revealed that ET-1 increases expression of CHSY1 through transactivation of EGF and TGF-β receptors.

**Conclusion:**

Transactivation through the EGF receptor mediated by phospho-ERK1/2 leads to expression of CHSY1 protein. EGF receptor transactivation by ET-1 is shown for the first time, to be dependent on NOX enzymes.

## Introduction

Based on the "response to retention hypothesis"stating, atherosclerosis is commenced by atherogenic lipoprotein entrapment by proteoglycan in the vessel wall. This is one of the crucial causes of atherosclerosis early stage. Proteoglycans are highly glycosylated proteins, consisting of core protein, which is covalently linked to glycosaminoglycan (GAG) chain. GAG chain is negatively charged due to the sulfate and carboxylic acid groups of chondroitin/dermatan sulfate (CS/ DS) chains. Therefore low density lipoprotein (LDL) particle (apo B100 has positively charged amino acid residues) interact with negative GAG chain results in retention of LDL in intima space of artery wall. Due to interactions with extracellular matrix (ECM) proteoglycan, LDL retention in the extracellular space of the arterial increases the chance of its oxidation and accumulation, promoting atherosclerosis (1, 2). Studies have shown that an increasing GAG chain length association with lipid binding affinity results in LDL retention in vascular wall and atherosclerosis development (3, 4). 

Chondroitin sulfate synthase 1 (CHSY1) is one of the family of GAG synthesizing enzymes involved in hyper elongation of GAG chain (5, 6). Previous atherosclerosis mouse model studies have demonstrated that GAG synthesizing enzymes genes elevated expression is correlated with increase in the GAG chain length and lipid deposition in vascular wall (3, 7).

Several growth factors have been shown to mediate alteration and modification (hyper-elongation) of proteoglycan structure via regulated expression of GAG bio-synthesis enzymes in the vascular smooth muscle cells (VSMCs) (8, 9). Endothelin-1 (ET-1) is a novel vasoconstrictor peptide, a 21 amino acid peptide that was produced by endothelial cells in the vascular wall (10, 11). Studies have revealed that ET-1 level increases in cardiovascular diseases such as atherosclerosis (12). Ballinger et al. (13) reported that ET-1 mediated elevation in radiosulfate incorporation secreted proteoglycan and induced proteoglycan synthesis in the VSMCs. ET-1 receptors (type A and type B) belong to G protein coupled receptors (GPCRs) family. GPCRs are seven transmembrane cell surface receptors which mediate pathophysiological cellular response through three signaling pathways. First, in classical pathway, GPCR agonists cause conformational changes in receptor that leads to downstream signaling activation. Second is β-arrestin scaffold pathway (14, 15) and third is transactivation pathway; transactivation signaling pathway was initially described by Daub et al. (16) who reported that GPCR agonists such as angiotensin II (Ang II) lead to activation and phosphorylation of epidermal growth factor receptor (EGFR) resulting in phosphorylation of downstream signaling mediators such as extracellular signal-regulated kinases (ERKs). GPCR agonists such as ET-1 and thrombin mediated TGF-β receptor type I (TβRI) activation by C-terminal phosphorylation of smad2 (pSmad2) induction, which is immediately TβRI downstream regulation that also, followed by increasing proteoglycan synthesis (14, 17, 18).

Chen et al. (19) reported that ET-1 mediated EGFR transactivation stimulated NADPH Oxidase (NOX), which produced reactive oxygen species (ROS) in different cell types such as cardiac fibroblasts. ROS generation is one of the critical components of the ET-1 signaling pathway. ROS activates and regulates numerous signal transduction cascades and gene expression in VSMC stimulated by ET-1, Thrombin, and Ang II. NOX enzymes especially produce ROS through acting as an electron donor to oxygen molecule in the superoxide anion formation. NOX1 is important in atherosclerosis pathogenesis. And, it is expressed in endothelial cells and VSMCs (20-22). 

In this study, we tested hypothesis that ET-1 could increase CHSY1 enzyme level through transactivation of EGF receptor and whether NOX is involved in this transactivation signaling pathway. The results revealed that ET-1 mediated p-ERK1/2 via EGFR transactivation. ET-1 from transactivation of two receptors (EGFR and TGFR) increased the expression of CHSY1 protein and NOX as an important mediator involved in this signaling pathway.

## Materials and Methods

This study was approved by the Research Committee and The Ethical Committee of The Ahvaz Jundishapur University of Medical Sciences (IR.AJUMS. REC.1395.575).

### Methods and Materials

In this experimental study, Dulbecco’s Modified Eagle Medium (DMEM F12), trypsin EDTA 0.025%, penicillin streptomycin, and fetal bovine serum (FBS) were purchased from Gibco (Invitrogen, Carlsbad, USA). SB431542, Tyrphostin AG 1478, bosentan, transforming growth factor β (TGF-β), ET-1 (Sigma, USA, cat no: E7764), EGF, Tween 20, bovine serum albumin (BSA), and dimethyl sulfoxide (DMSO) were from Sigma Alderich (USA). Bicinchoninic acid (BCA) protein assay kit was purchased from Pars Tous (Iran). Polivinilydene difluoride (PVDF) membrane was purchased from Roche Diagnostic (Mannheim, Germany). Tris, Glysine, SDS, TEMED, and acrylamide were from Merck (Germany). Phospho p44/42 MAPK (ERK1/2) (Thr202/Tyr204) Rabbit monoclonal antibody (Cell signaling: 4370S) and HRP- anti-rabbit IgG- peroxidase were from Cell Signaling Technology (Beverly, MA, USA). Anti GAPDH antibody and anti CHSY1 antibody (Abcam, USA, cat no: 153813) were purchased from Abcam (Cambridge, MA, USA). Chemiluminesence ECL detection kit was from BIO-RAD (Hercules, CA, cat no: 1705061).

### Cell culture

Cell culture Human VSMCs were grown in DMEM-F12 supplemented with 10% FBS and 1% Penicillin-Streptomycin at 37˚C in 5% CO_2_ followed by 24 hours starvation in DMEM-F12 with 0.1% FBS before treatments

### Western blotting

After treatment with various agents, the cells were washed twice with cold PBS and lysed in 75 μl RIPA buffer), and all cell lysates were collected. Protein concentration was measured by BCA assay. Equal amounts of protein (50 μg) were loaded and resolved on 10% SDS-polyacrylamide gel electrophoresis, transferred to PVDF membrane, and then incubated overnight at 4˚C with anti p-ERK1/2 (Thr202/Tyr204) antibody (1:2000) and anti CHSY1 antibody (1:1000), followed by HRP-anti rabbit IgG (1:10000), secondary antibody, for 1 hour at room temperature, and protein band visualized by ECL. To determine equal loading of proteins, the membrane was striped and re-incubated with GAPDH polyclonal antibody (1:2500) followed by a secondary HRP-conjugated antibody (1:10000) followed by development with enhanced chemiluminescence ECL clarity (Bio-Rad, Hercules, CA) and bands were visualized with BioRad ChemiDoc. Image J software was used to describe the intensity of protein bands (23).

### Statistical analysis

Results are presented as the mean ± standard error of the mean (SEM) of three independent experiments. Statistical analysis was performed by one way ANOVA test, and P<0.05 and P<0.01 were considered significant.

## Results

### EGF-mediated phosphorylation of ERK1/2 (Thr202/ Tyr204) in VSMCs

**Fig.1:**
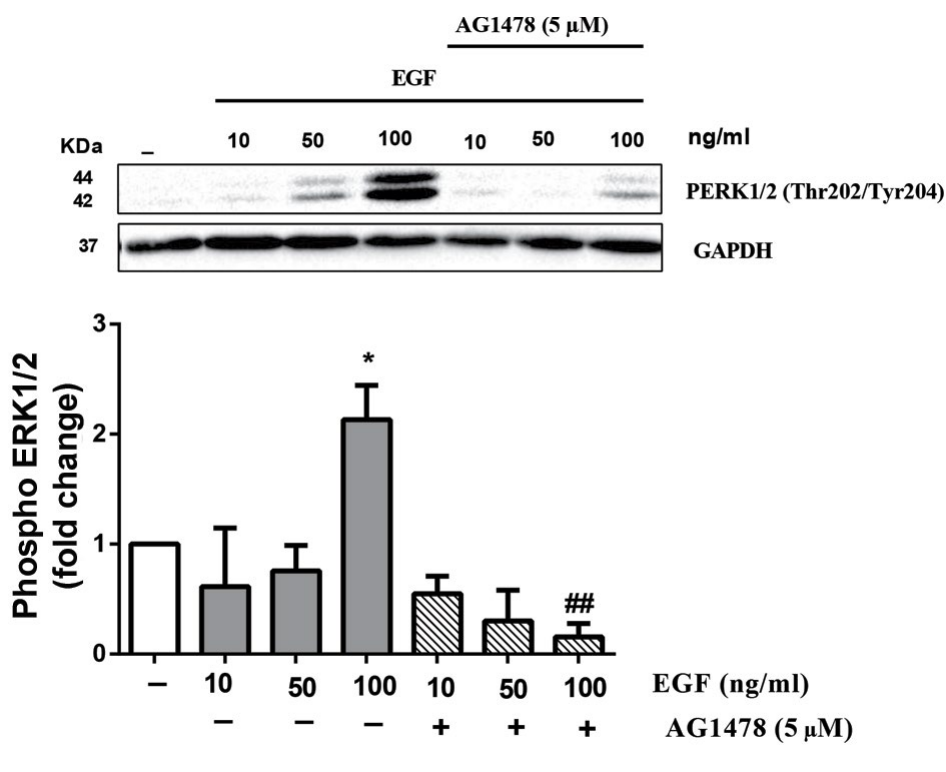
EGF induced ERK1/2 (Thr202/Tyr204) phosphorylation via EGFR. VSMCs were treated with AG1478 (5 µM) for 30 minutes before adding EGF. After 5 minutes, stimulation with EGF cell lysates were immuneblotted with anti ERK1/2 (Thr202/Tyr204) followed by HRP-conjugated rabbit IgG secondary antibody. The band intensity of three independent blots were measured by densitometric quantitation. *; P<0.05 control vs. EGF, ##; P<0.05 antagonist vs. EGF, EGFR; Epidermal growth factor recptor, ERK; The extracellular signal‑regulated kinases, VSMCs; Vascular smooth muscle cell, and HRP; Horseradish peroxidase.

In order to verify whether ERK1/2 is phosphorylated by EGF through its receptor (EGFR), VSMCs were treated with increasing dose of EGF, respectively, 10, 50, 100 ng/ml. We observed EGF at 100 ng/ml dose stimulates phosphorylation of ERK1/2 significantly (P<0.05) at 5 minutes. Also, AG1478 (5 µM), the antagonist of EGFR, inhibited EGF-stimulated ERK1/2 phosphorylation (P<0.05, Fig .1) which showed EGF through its receptor induced ERK1/2. 

### ET-1 caused phosphorylation of ERK1/2 (Thr202/ Tyr204) in VSMCs

**Fig.2:**
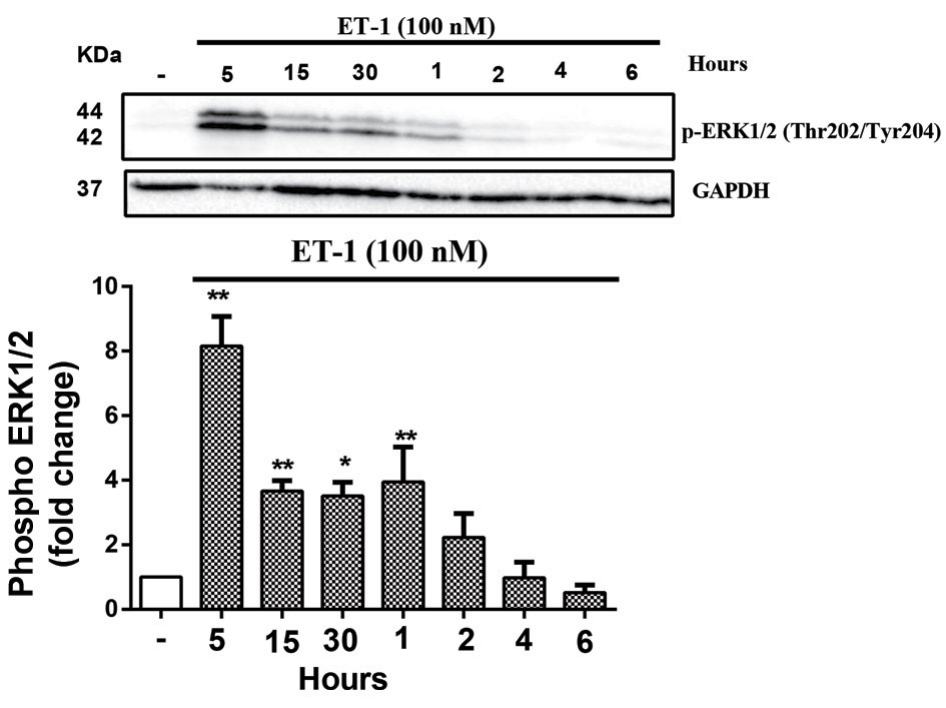
ET-1 mediated phosphotylation of ERK1/2 (Thr202/Tyr204) in the VSMCs. VSMCs were treated with ET-1 (100 nM) for 6 hours. Cell lysates were immunoblotted with anti ERK1/2 (Thr202/Tyr204) followed by HRP-conjugated rabbit IgG secondary antibody. The band intensity of three independent blots were measured by densitometric quantitation. *; P<0.05, **; P<0.01 control vs. ET-1 treated group, ET-1; Endothelin-1, ERK; The extracellular signal‑regulated kinases, VSMC; Vascular smooth muscle cell, and HRP; Horseradish peroxidase.

To examine whether ET-1 induced ERK1/2, VSMCs were treated with ET-1 (100 nM) at different time points respectively, 5, 15, 30 minutes and 1, 2, 4, 6 hours. Our results showed that maximum ERK1/2 phosphorylation was at minute 5 (P<0.01, Fig .2).

### ET-1 mediated ERK1/2 (Thr202/Tyr204) phosphorylation increase via both of ET receptors and EGFR transactivation 

**Fig.3:**
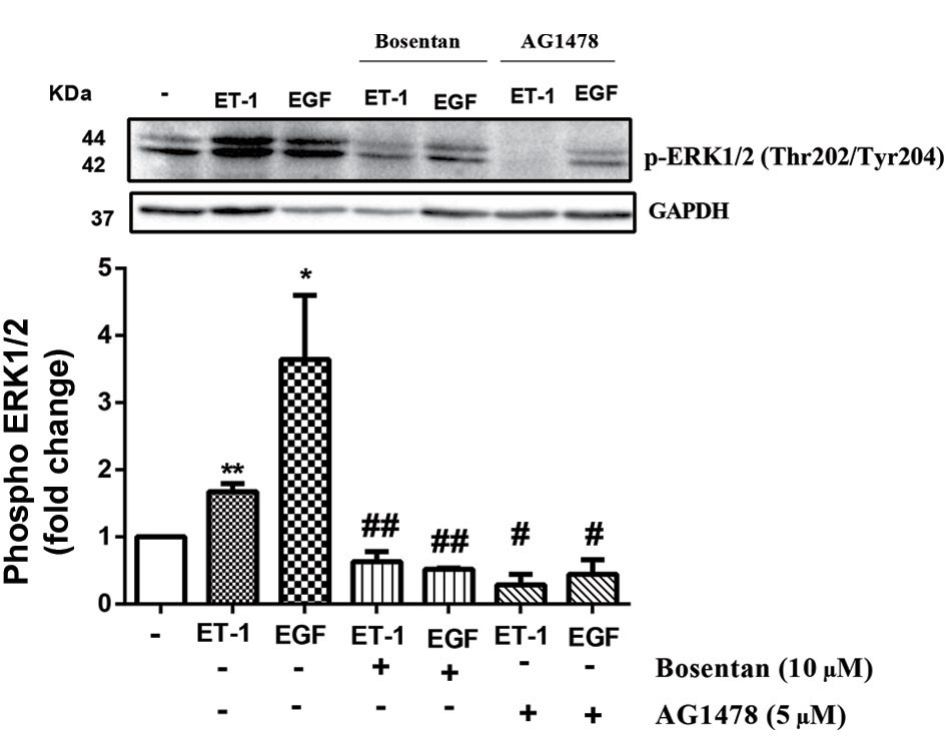
ET-1 stimulation increased phosphorylation of ERK1/2 (Thr202/ Tyr204) via its receptor and receptor tyrosine kinase EGF (EGFR) Transactivation. VSMCs were treated with bosentan (10 µM) and AG1478 (5 µM) for 30 minutes before stimulation with EGF (100 ng/ml) or ET-1 (100 nM) for 5 minutes. Cell lysates were immunoblotted with anti ERK1/2 (Thr202/Tyr204) followed by HRP-conjugated rabbit IgG secondary antibody. The band intensity of three independent blots were measured by densitometric quantitation. *; P<0.05, **; P<0.01 control vs. growth factor treated group, #; P<0.05, ##; P<0.01 antagonist vs. growth factor treated group, ET-1; Endothelin-1, ERK; The extracellular signal‑regulated kinases, EGF; Epidermal growth factor, VSMC; Vascular smooth muscle cell, and HRP; Horseradish peroxidase.

To investigate ET-1 mediated increase in p-ERK1/2 via its receptor, the cells were treated in the presence and absence of bosentan (10 µM), the antagonist of ET-1 receptors (ETA and ETB receptors), for 30 minutes prior to treatment with ET-1 (100 nM). ET-1 had about 2-fold increase in the ERK1/2 phosphorylation (P<0.01) at minute 5. Bosentan completely inhibited the stimulatory effect of ET-1 on p-ERK1/2 (P<0.01). To explore the EGFR role on ERK1/2 phosphorylation by ET-1, ET-1 and EGF were stimulated in the presence of AG1478. AG1478 (P<0.01) abolished ET-1 stimulated phosphorylation of ERK1/2 (Fig .3). 

### NOX acted as a mediator of ET-1 stimulated of p-ERK1/2 (Thr202/Tyr204) through EGFR

**Fig.4:**
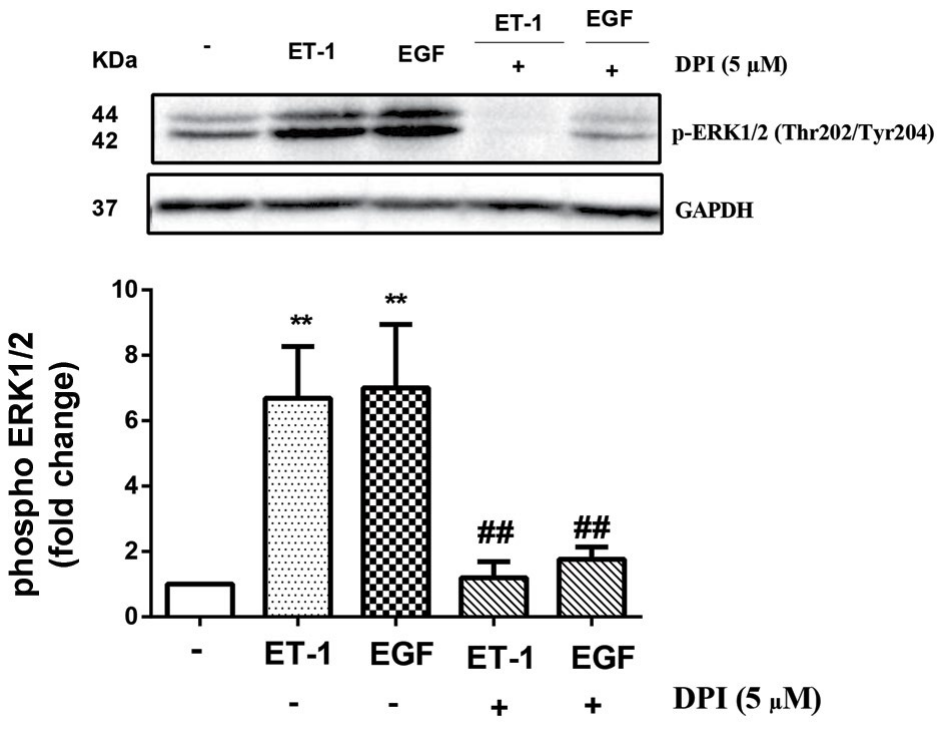
ET-1 mediated phosphotylation of ERK1/2 (Thr202/Tyr204) in the VSMCs. VSMCs were treated with ET-1 (100 nM) for 6 hours. Cell lysates were immunoblotted with anti ERK1/2 (Thr202/Tyr204) followed by HRPconjugated rabbit IgG secondary antibody. The band intensity of three independent blots were measured by densitometric quantitation. *; P<0.05, **; P<0.01 control vs. ET-1 treated group, ET-1; Endothelin-1, ERK; The extracellular signal-regulated kinases, VSMC; Vascular smooth muscle cell, and HRP; Horseradish peroxidase.

To evaluate the implication of NOX in phosphorylation of ERK1/2 caused by ET-1 or EGF, DPI (5 µM), the inhibitor of NOX, was used for 2 hours prior to the treatment with ET-1 or EGF. DPI completely attenuated the stimulatory effect of ET-1 and EGF (P<0.01) on p-ERK1/2 (Fig .4).

### ET-1, TGF-β, and EGF stimulation invoked an increase in the level of CHSY1 protein

**Fig.5:**
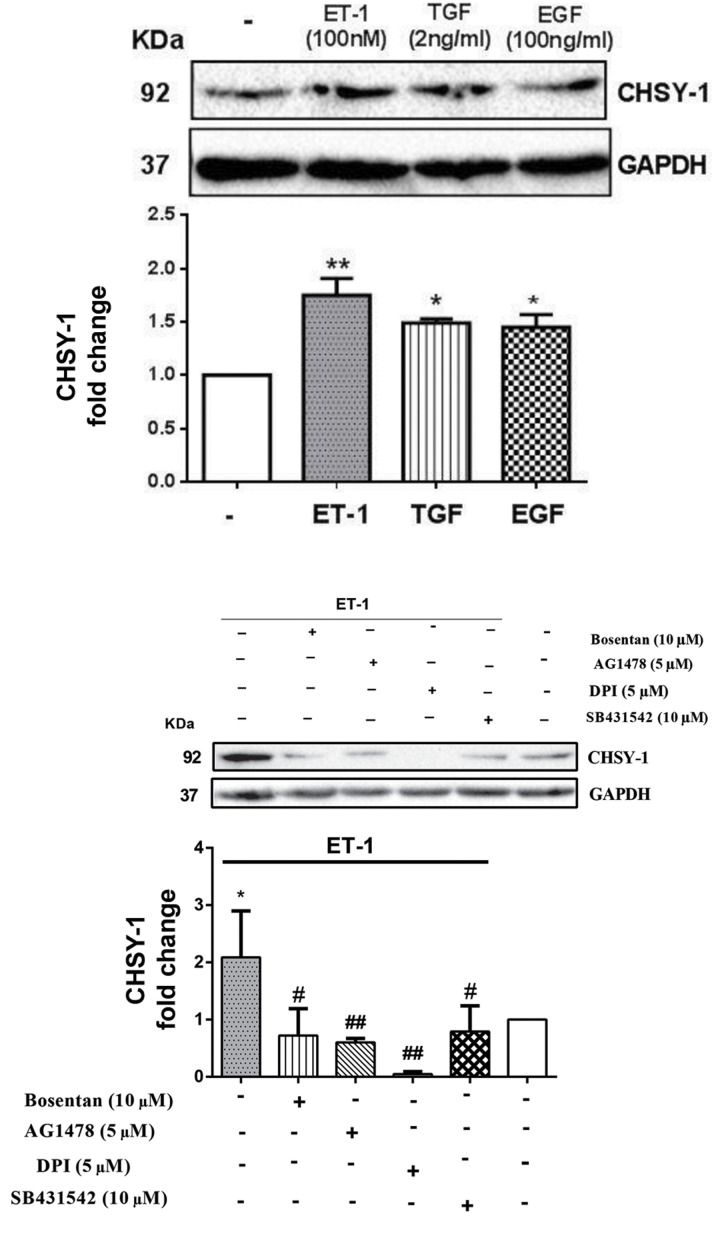
ET-1, TGF-β, and EGF increased CHSY-1 mediated by NOX. **A.** ET-1, TGF-β, and EGF mediated increase CHSY1 protein level. VSMCs were treated with ET-1 (100 nM), TGF-β (2 ng/ml), and EGF (100 ng/ml) for 24 hours. Cell lysates were immunoblotted with anti CHSY1 antibody followed by HRP-conjugated rabbit IgG secondary antibody. The band intensity of three independent blots were measured by densitometrics quantitation. *; P<0.05 and **; P< 0.01 control vs. growth factor treated group. **B.** ET-1 mediated increases CHSY1 protein level by transactivation of EGFR and TβRI, and NOX is a mediator of this signaling pathway. VSMCs were treated with ET-1 (100 nM) in the presence of each of the following inhibitors: Bosentan (10 µM), SB431542 (10 µM), and AG1478 (5 µM) for 30 minutes; DPI (5 µM) for 2 hours before treatment with ET-1 (100 nM) for 24 hours. Cell lysates were immunoblotted with anti CHSY1 antibody followed by HRP-conjugated rabbit IgG secondary antibody. The band intensity of three independent blots were measured by densitometrics quantitation. *; P<0.05 control vs. ET-1, #; P<0.05, ##; P<0.01 antagonist vs. growth factor treated group, ET-1; Endothelin-1, TGF-β; Transforming growth factor-β, EGF; Epidermal growth factor, VSMC; Vascular smooth muscle cell, DPI; Diphenyleneiodonium, and HRP; Horseradish peroxidase.

To confirm the stimulatory effect of ET-1, TGF-β, and EGF on the CHSY1 protein level, VSMCs were exposed to ET-1 (100 nM), EGF (100 ng/ml), and TGF-β (2 ng/ ml) for 24 hours. About 2-fold increase was observed on the level of CHSY1protein following of ET-1 addition (P<0.01). Also, VSMCs treated with EGF and TGF-β showed an increase on the level of this protein (P<0.05, Fig .5A).

### ET-1 induced increase level of CHSY1 protein via transactivation of EGFR and TβRI, a process that also mediated by NOX

It is important to show that which receptors or pathways are involved in ET-1 induced CHSY1 protein synthesis. Also, the CHSY1 protein level was upregulated following ET-1 stimulation (100 nM, for 24 hours), and this response was blocked in the presence of bosentan (P<0.05), indicating the specific role of ET-1 receptors which mediated increase in CHSY1 protein. 

SB431542 (TβRI inhibitor) was used to assess ET-1 as well through transactivation of TβRI, increasing the level of CHSY1 protein. Our result showed that SB431542 (10 µM) reduces the CHSY1 protein induced by ET-1 (P<0.05). 

To confirm EGFR transactivation by ET-1 increased CHSY1 protein level as a target protein; the role of EGFR was also examined in this transactivation pathway by utilization of AG1478. We obseved ET-1 induced increase in CHSY1 protein level is attenuated in the presence of AG1478 (P<0.01). 

ET-1 exerts its effect to induce expression of gene such as CHSY1 protein through transactivation of both EGFR and TβRI. To evaluate the role of NOX as a mediator in this pathway, we used DPI (5 µM) for 2 hours before treatment with ET-1. DPI significantly reduced ET-1 stimulatory effect on the level of CHSY1protein (P<0.01, Fig .5B).

## Discussion

The role of ET-1 transactivation of EGFR and TβRI was studied on phosphorylation of ERK1/2 and production of GAG synthesizing enzyme, CHSY1. ET-1 stimulates phosphorylation of ERK1/2 and increases the level of CHSY1 protein via EGFR and TβRI, and NOX has a crucial role in this pathway.

EGF induced p-ERK1/2 and also stimulated VSMC by ET-1, that resulted in the ERK1/2 phosphorylation, which is consistent with the data observed by Yogi in VSMCs (24). Bosentan, a specific antagonist of ET receptors, attenuated ERK1/2 phosphorylation, that verified the specific role of ET-1 receptors in ERK1/2 phosphorylation. Daub et al. (16) presented the first evidence of GPCR transactivation on protein tyrosine kinase receptor (PTKR) in rat fibroblasts. They proved that transactivation of PTKR such as EGFR by GPCR agonists like ET-1, thrombin, and lysophosphatidic acid mediated ERK1/2 phosphorylation and activation of downstream signaling pathways. ERK1/2 plays an important role in the GAG hyper-elongation in VSMCs (3). We found that AG1478 (EGFR antagonist) reduced the ET-1 effect on p-ERK1/2, suggesting that p-ERK1/2 level increase by ET-1 mediation is dependent on EGFR transactivation. GPCRs transactivated EGFR through two major mechanisms: ligand dependent and ligand independent pathways. In ligand dependent EGFR transactivation pathway, activation of EGFR depends on the binding of active ligands such as heparin binding EGF like growth factor (HB-EGF) which comes from the cleavage. This cleavage is mediated by a disinterring and a metalloproteinase (ADAM), a group of matrix metalloproteinase (MMP). Activated MMPs cleavage HB-EGF ligand and release EGF into extracellular space that resulted in exposure to the EGF receptor. And, this causes the dimerization and stimulation of EGFR. Further, the activated receptor is able to stimulate ERK MAP kinase pathway, the downstream signaling cascade. In the ligand independent mechanism, EGFR transactivation via GPCR agonists occurs through activation of several second messengers such as Ca^2+^, ROS, and Src tyrosine kinase (15, 25, 26). ET-1 has been shown to be associated with the elevation of NOX activity and consequently the production of ROS generation in the human VSMCs (22), which has a main role in the majority of GPCR signaling pathways (19). Similarly, assessing the NOX role in EGFR transactivation mediated by ET-1, we observed that it causes ERK1/2 phosphorylation in VSMCs. It was found that DPI decreased apparently p-ERK1/2 induction by ET-1 and EGF, confirming that NOX has an important role in ET-1 transactivation of EGFR which brings about ERK1/2 phosphorylation. This is also consistent with previous studies, reporting that GPCR agonists, including Ang II and thrombin mediated EGFR transactivation are dependent on NOX in VSMCs (27, 28).

Based on studies, retention of LDL on hyper-elongated GAG chains on proteoglycans such as biglycan results in increased foam cell formation and progression of arterial wall atherosclerosis (1). CSs are widely prevalent sulfated carbohydrates on cell surfaces and in the ECM. CS belongs to the GAG family that consists of disaccharide units containing glucuronic acid and N-acetylgalactosamine residues. It is synthesized as a CS proteoglycan by attaching linear CSs to Ser residue in the core protein. Also, CHSY1, glycosyltransferases enzyme, is responsible for biosynthesis of chondroitin and dermatan sulfate GAG. It is shown that increased sulfated GAG, mainly CSc, enhances CHSY1 transcriptional activity (29). Anggraeni et al. (7) showed that 8 weeks high fat diet feeding increased plaque development and markedly elevated level of CHSY1 mRNA expression in a mouse model. The current results demonstrated that ET-1 enhanced CHSY1 level expression in VSMCs. Also, EGF and TGF-β, both which are known to be involved in the atherosclerosis development, up-regulates CHSY1 level. Recent studies demonstrated that TGF-β mediated proteoglycan synthesis and up-regulated mRNA expression of CHSY1 in retinal choroidal endothelial cell and VSMCs (8, 30). Also Kamato et al. (31) demonstrated that EGF treatment of VSMCs increased GAG length and induced mRNA expression of CHSY1 through downstream intermediate ERK1/2 which is blocked by AG1478. Rostam et al. (30) reported that increased protein level of CHSY1 and other GAG synthesizing enzymes were associated with elevated induction of their mRNA expression. It has been previously reported that thrombin transactivated EGFR and TβRI that both of them are involved in phosphorylation of Smad2 linker region and increase of CHSY1 enzyme mRNA expression (23, 31). It was found that ET-1, stimulated increased level of CHSY1 protein that were inhibited by the ET-1 receptors antagonist. This issue suggested that the response is mediated through ET-1 receptors. Also, ET-1 was reported to cause enhanced proteoglycan synthesis and GAG chain hyper-elongation (11-13). In addition, CHSY1 is induced by ET-1 and also, decreased by SB431542 and AG1478. It is suggested that the ET-1 stimulation of CHSY1 is mediated by transactivation of EGFR and TβR1. To address the role of NOX on mediating the transactivation of EGFR and TβRI, we investigated the level of CHSY1 protein in the presence of DPI, NOX antagonist,; it was confirmed that NOX is involved in ET-1 transactivation of EGFR, and TβR1 mediated the elevation of CHSY1 expression.

The strength of the current study is the involvement of NOX in ET-1 transactivation of both EGFR and TβRI that mediated elevation on CHSY1 protein level.

## Conclusion

It was demonstrated that ET-1 increased the level of p-ERK1/2, and this response was attenuated by AG1478, suggesting that ET-1 causes ERK1/2 phosphorylation, mediated through transactivation of EGFR. Also, this mechanism is dependent on NOX signaling. It was indicated that ET-1 mediated the elevated level of CHSY1 protein, which is implicated in hyperelongation of GAG chain through transactivation of EGFR and TβRI. Moreover, TGF-β and EGF mediated the elevation of CHSY1protein level, which was inhibited by NOX antagonist.
